# CUSP9* treatment protocol for recurrent glioblastoma: aprepitant, artesunate, auranofin, captopril, celecoxib, disulfiram, itraconazole, ritonavir, sertraline augmenting continuous low dose temozolomide

**DOI:** 10.18632/oncotarget.2408

**Published:** 2014-08-29

**Authors:** Richard E. Kast, Georg Karpel-Massler, Marc-Eric Halatsch

**Affiliations:** ^1^ IIAIGC Study Center, Burlington, VT, USA; ^2^ University of Ulm, Department of Neurosurgery, Albert-Einstein-Allee 23, Ulm, Germany

**Keywords:** chemotherapy, glioblastoma, growth factors, CUSP9*

## Abstract

CUSP9 treatment protocol for recurrent glioblastoma was published one year ago. We now present a slight modification, designated CUSP9*. CUSP9* drugs- aprepitant, artesunate, auranofin, captopril, celecoxib, disulfiram, itraconazole, sertraline, ritonavir, are all widely approved by regulatory authorities, marketed for non-cancer indications. Each drug inhibits one or more important growth-enhancing pathways used by glioblastoma. By blocking survival paths, the aim is to render temozolomide, the current standard cytotoxic drug used in primary glioblastoma treatment, more effective. Although esthetically unpleasing to use so many drugs at once, the closely similar drugs of the original CUSP9 used together have been well-tolerated when given on a compassionate-use basis in the cases that have come to our attention so far. We expect similarly good tolerability for CUSP9*. The combined action of this suite of drugs blocks signaling at, or the activity of, AKT phosphorylation, aldehyde dehydrogenase, angiotensin converting enzyme, carbonic anhydrase -2,- 9, -12, cyclooxygenase-1 and -2, cathepsin B, Hedgehog, interleukin-6, 5-lipoxygenase, matrix metalloproteinase -2 and -9, mammalian target of rapamycin, neurokinin-1, p-gp efflux pump, thioredoxin reductase, tissue factor, 20 kDa translationally controlled tumor protein, and vascular endothelial growth factor. We believe that given the current prognosis after a glioblastoma has recurred, a trial of CUSP9* is warranted.

## INTRODUCTION

One year ago, we published the original CUSP9 treatment protocol for recurrent glioblastoma [1]. CUSP9, Comprehensive Undermining of Survival Paths, was an attempt to block growth facilitating or growth driving signaling systems that have been identified as active in glioblastoma. To develop CUSP9 we found nine re-positioned (re-purposed), already-marketed drugs that had evidence supporting their ability to inhibit one or more of the identified glioblastoma growth and cell survival pathways [1]. CUSP9 has been well-tolerated in the patients that have come to our attention who have been given CUSP9 on a compassionate-use basis. There is no word yet on effectiveness. We present here an update on the rationale and some minor changes to this protocol, designated CUSP9*.

The basic idea behind CUSP9 treatment was detailed in the original document [1] and has not changed for the current CUSP9*. Briefly, given the impasse we have been in since temozolomide introduction as standard initial treatment in 2005 [2] we developed a conceptually new approach. Instead of focusing on finding new cytotoxic drugs or variations combining traditional cancer chemotherapeutic drugs we looked at both native growth-promoting systems and the many escape paths that are mobilized by glioblastoma cells during exposure to current cytotoxic drugs like temozolomide. We then surveyed the research literature looking for already-marketed non-cancer treatment related drugs for which we have data or evidence that that they might block or inhibit one of these identified cytotoxicity circumvention pathways. Using low likelihood of adding to patient side effect burden, good quality of life (QOL) maintenance, and good clinical experience with the drug in question as additional selection criteria, we arrived at nine drugs to augment temozolomide's anticancer activity: aprepitant, artesunate, auranofin, captopril, Cu gluconate, disulfiram, ketoconazole, nelfinavir, and sertraline [1].

**Fig. 1:**
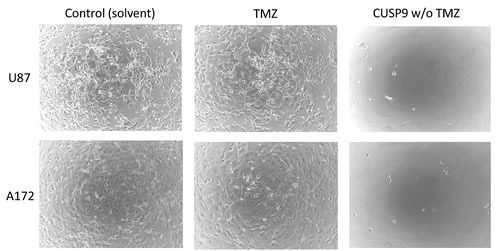
*In vitro* activity of CUSP9 drugs compared to that of temozolomide alone The cell-killing effects of CUSP9 without temozolomide versus temozolomide alone on glioblastoma cell lines *in vitro*. Two different glioblastoma cell lines were incubated with CUSP9 compounds without temozolomide (TMZ) or with TMZ alone at the respective concentrations commonly achievable in human plasma or cerebrospinal fluid (if the latter data were available). Control cells were treated with the corresponding amount of solvent only. Microphotographs were taken after 7 days of continuous exposure to CUSP9, TMZ, or solvent respectively. While glioblastoma cells grew rapidly under control conditions, all CUSP9 without TMZ-treated cells had died. In comparison, TMZ conferred a markedly weaker inhibition of cell growth.

In Fig. 1. the profound effect of the original CUSP9 drugs without temozolomide can be seen. Deep cytotoxicity occurs in glioblastoma cell lines at drug levels expected *in vivo*. The new, slightly modified CUSP9* shows even greater cytotoxicity to glioblastoma cell lines at even lower concentrations. The deep *in vitro* cytotoxicity of both CUSP9 and CUSP9* drugs to glioblastoma cell lines is the subject of a separate publication.

Since CUSP9 publication, the European Medicines Agency has delisted ketoconazole, and the manufacturer of nelfinavir has stopped production. Related drugs were therefore substituted in CUSP9*, itraconazole for ketoconazole, ritonavir for nelfinavir. As disulfiram achieves much of its anticancer effectiveness only after one of its metabolites chelates copper, we added copper gluconate to CUSP9. However, disulfiram derivatives chelate copper already in the stomach [references given in Table 4.], negating need for exogenous copper. In CUSP9* therefore, copper gluconate was deleted. In its stead, celecoxib adds further dimensions to our targeting of the many “complementary or redundant pathways” [3] glioblastoma uses to grow and evade our cytotoxicity attempts. CUSP9* therefore consists of simultaneous administration of aprepitant, artesunate, auranofin, captopril, celecoxib, disulfiram, itraconazole, sertraline, and ritonavir. They are designed to be administered with low-dose, uninterrupted, daily temozolomide. Repositioning already-marketed drugs to block survival and growth paths in glioblastoma remains the watchword.

None of the 22 studies of new cytotoxic drugs, or cytotoxic drug combinations for recurrent glioblastoma reporting in 2012 gave meaningful clinical benefit [1]. We now report similarly sad results for the 27 studies reporting in 2013. Twelve of these 2013 studies are listed in Table 1. Since entry conditions were different for these twelve studies no comparative conclusion can or should be drawn from the differing OS, other than, clearly, no breakthroughs have been made. Not shown in Table 1 are 15 studies that were stopped early for futility, disastrous QOL deterioration, or studies where design vagarities didn't permit OS determination.

**Table 1:** Overall survival, OS, in 12 of the 27 clinical studies on new treatments for recurrent glioblastoma reporting in 2013 The remaining 15 studies could not be evaluated for OS but none seemed to show dramatic benefit or evidence that their numbers would be much different from the 12 listed here. It is important not to conclude from the differing OS in this table that one treatment might be different or better than another. Study entry criteria, previous treatments, and other variables make close comparisons between these studies impossible. What we can conclude is that OS is short, glioblastoma is an aggressive disease and better treatments are needed

Drugs or treatment	median OS [months]	reference
Dose-dense temozolomide + tamoxifen	17	290 19
Re-irradiation	13	291 3
bevacizumab and temsirolimus	4	292 4
open laser ablation	11	293 5
temozolomide 100 mg/m2/day x 21 days, 7 days off, repeated	12	294 6
erlotinib and sorafenib	6	295 7
temozolomide and lapatinib	6	296 8
continuous low dose temozolomide 50 mg/m^2^/day	7	7 9
nintedanib	6	297 10
bevacizumab and erlotinib	10	298 11
sunitinib	9	299 12
autologous vaccine+ HSP96	12	300 13

Four additional guiding principles for CUSP9* formulation remain as for CUSP9:

A. Careful attention to using drugs that have a low likelihood of increasing patient side effect burden, a low likelihood of interfering with each other, and a research database allowing reasonable expectation for anti-glioblastoma effects.

B. Importance of having a broad, comprehensive approach, blocking potential as well as actual cytotoxicity escape paths. We aimed to dismantle every glioblastoma cell defense mechanism we could identify for which we had already-marketed inhibitors that also have low inherent risk by themselves and don't have predictable areas of interference with each other. Although designed to be used with low dose continuous temozolomide 50 mg/m^2^/day by mouth, recent research is showing anti-glioma activity of the CUSP9* drugs even without temozolomide. This data will be discussed below in the individual drugs' sections.

**Fig. 2:**
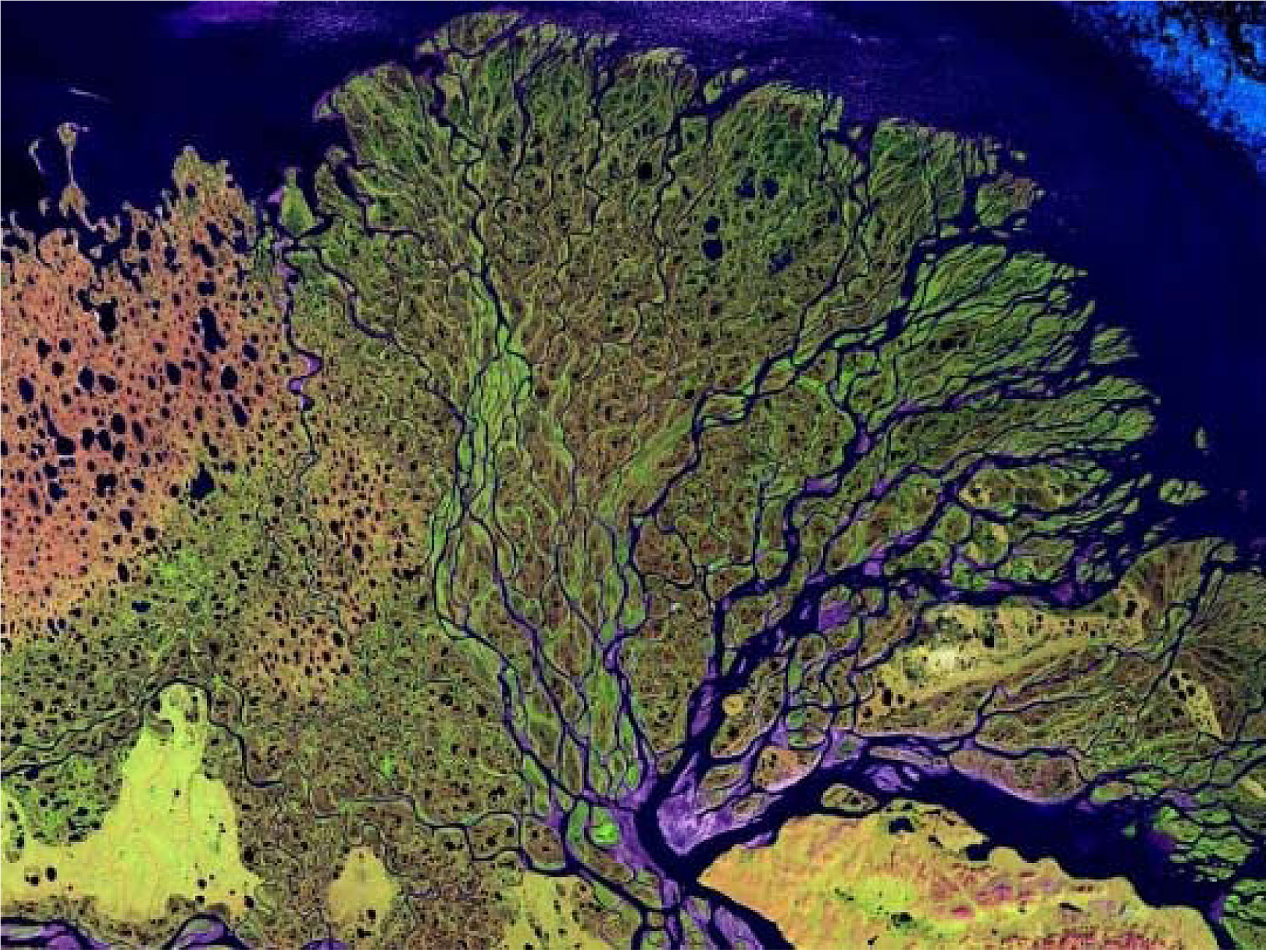
A satellite image of a river flowing into the Arctic Ocean, illustrating what we call the “Nile Distributary Problem” where multiple distributaries develop that can cross-cover, maintaining total flow should the flow at one distributary become blocked.

C. When one or several growth paths are blocked, a cell, and particularly a cancer cell, shifts reliance to other parallel or compensating paths that have not been blocked. We term this the “Nile Distributary Problem” in that if one distributary at the delta is blocked, total flow into the sea remains unaltered. The unblocked distributaries take up the water that would have flowed through the blocked distributary as can be imagined from satellite image in Fig. 2. Representative quotes in Table 2. are from various authors referring to this phenomenon in cancer growth using different words to discribe this idea. Hence, need for a CUSP-type broad-net protocol.

**Table 2:** Intratumoral spatial and temporal heterogeneity Selected recent quotes on the strand of thinking in oncology from the last two years, the principles upon which CUSP9* was developed. Words outside of quotation marks are our addition but we believe in the spirit in which the quoted authors meant their comments. CSC = cancer stem cell; GBM = glioblastoma

Effective “treatment of recurrent GBM includes combination strategies with agents that target complementary or redundant pathways” [3].
Writing of a study of renal cell carcinoma…“Gene-expression signatures of good and poor prognosis were detected in different regions of the same tumor…..[there was] extensive intratumor heterogeneity” [301]
There is “bewildering…heterogeneity” over time following the same tumor and “within individual tumor biopsies [that are] spatially separated” [302].
Writing of head/neck squamous cell carcinoma “intratumor heterogeneity showed that a single biopsy may not represent the entire mutational landscape” [303.].
Reviewing plasticity and CSC “non-CSCs can reacquire a CSC phenotype” [304.].
Writing of cancers generally…there are multiple” dynamic interrelationship[s] between intratumoral cell subpopulations…[that have] clear clinical significance” [305.].
Writing of aggressive breast cancer…“coexistence of tumor cells with different phenotypic traits within a primary tumor” with “branched …[bidirectional] …evolutionary tumor growth” concluding “primary tumors are ecosystems of evolving clones with different spatial and temporal distributions” that are mutually reinforcing [5].
In offering their data and interpretation on why inhibiting growth factor receptor inhibition has not been effective in prolonging glioblastoma survival…”growth factor receptor/PI3K/AKT pathway is complex and nonlinear having many inputs from other pathways (cross covering paths compensating for a particular block), multiple sites of feedback regulation (both positive and negative), and a large number of downstream effectors “[306] (Our comments in parentheses.)

D. Addressing tumor microregional heterogeneity in time and space, the “multifaceted heterogeneity” of glioblastoma both between different tumors of similar H&E histology and within an individual tumor both in space and over time [3, 4]. A recent study of aggressive human breast cancers by Costa et al is exemplary of this phenomenon [5] that applies to glioblastoma. They demonstrated marked micro-regional heterogeneity within a single tumor mass with respect to a particular proteinase, ADAM-23, showing further that ADAM-23 positive and ADAM-23 negative subpopulations had different but mutually supporting functions [5]. Heterotypic environments create growth vigor in that ADAM-23 negative cells alone are invasion-competent but proliferation-poor while ADAM-23 positive subpopulations are proliferation-competent, invasion-weak [5]. We look to this phenomenon of mutually reinforcing subpopulations with spatial and temporal intratumoral heterogeneity that is also unstable over time as a primary cause of previous chemotherapy failures.

Although the particular proteinase Costa et al studied might not be a universal or even an important feature of malignancies, we believe that the principle is paradigmatic of cancers. Heterotypic environments within a tumor, shifting over time and space is of extraordinary importance to our understanding of malignant growth. Many researchers have recently come to related conclusions on this matter, selected examples given in Table 2. Hence, we must cast a wide net with CUSP9*, multi targeting to match glioblastoma heterogeneity in time and space.

Also related to this aspect of CUSP9*, concordant in principle and thinking, was the recent COMBAT trial [6] where 74 children with various advanced, treatment-refractory cancers were treated with six drugs- low-dose daily temozolomide, etoposide, celecoxib, vitamin D, fenofibrate and retinoic acid (COMBAT regimen). Temozolomide and etoposide are traditional cytotoxic chemotherapeutic agents. The four ancillary drugs were designed to, as in CUSP9*, block survival paths. It was adequately tolerated enough and authors considered that their study had evidence enough of benefit in this heavily pretreated population to warrant further study [6].

### The CUSP9* drugs

Table 1. lists several studies on recurrent glioblastoma that reported in 2013. A summary of the CUSP9* drugs showing half-life, and several core rationale(s) for the individual drugs' inclusion in, and contribution to, CUSP9* is given in Table 3. Table 4. lists the CUSP9* drugs, P-450 engagements and their common side effects.

The choice of low dose continuous temozolomide [50 mg/m^2^/day by mouth] was made in view of the low side effect burden imposed by this regimen along with the absence of any other cytotoxic regimen demonstrating significantly longer overall survival (OS) [7, 8]. Note that one of these studies combined temozolomide with another of the CUSP9* drugs, celecoxib [8]. The authors noted that the combination “seems to have activity in recurrent glioblastoma without relevant toxicity” but their progression-free survival at 6 months was only 43% [8]. Clearly better is needed.

**Table 3:** Overview of CUSP9* drugs with circulating half-life and basic growth paths inhibited Breast cancer resistance protein, BCRP; Neurokinin-1, synonymous with 11 amino acid, 1.3 kDa peptide substance P; angiotensin converting enzyme, ACE reactive oxygen species, ROS, a term used to refer to any atom with an unpaired valence electron; Translationally controlled tumor protein, TCTP; matrix metaloproteinase -2, -9, MMPs; References are given in the drug's section in text

DRUG	T1/2	core survival pathway[s] inhibited…
ARTESUNATE ------------------ active metabolite dihydroartemisinin	< 1 hr ------ 1 hr	phosphoinositide 3-kinase, Akt, increases ROS, NF-κB activation, TNF-alpha, IL-6, TLR2, ------- Same
APREPITANT	10 hrs	NK-1 receptors
AURANOFIN	10 days	thioredoxin, increases ROS, STAT3
CAPTOPRIL	2 hrs	ACE, AT1 receptors, MMPs
CELECOXIB	9 hrs	COX-1 and -2, carbonic anhydrase -2 and -9
DISULFIRAM ----------------- active metabolite diethyldithiocarbamate	<2 hrs ---- 6 hrs	ALDH, increases ROS -------- same
ITRACONAZOLE	19 hrs	P-gp efflux transporters, BCRP, Hedgehog, 5-lipoxygenase
RITONAVIR	4 hrs	P-gp efflux transporters [weak], Akt, mTOR, cyclin D3, proteasome,
SERTRALINE	1 day	Akt, mTOR, TCTP

**Table 4:** CUSP9* drugs with hepatic P-450 engagements and expected side effect profile based on data and clinical experience with each drug when used individually * = Alcohol intolerance is not listed as side effect of disulfiram even though this is severe and universal because ALDH inhibition and consequent alcohol intolerance is the main and expected effect of disulfiram. References to the pharmacology and side effect profile of the individual drugs are given in the text. GFR, glomerular filtration rate; LFT, liver function tests;

DRUG	p-450 inhibited	metabolism by	common SE	rare SE	ref.
aprepitant	3A4	3A4	hiccups, asthenia, diarrhea	none	309, 311, 316
artesunate	2D6 slight	2A6 slight	altered taste	neutropenia ?	307
auranofin	none known	unknown	diarrhea, rash,	stomatitis, neutropenia thrombocytopenia	308, 310 312
captopril	none known	unknown	orthostatic hypotension, cough	none	322
celecoxib	2D6	2C9 [3A4 slight]	headache, edema, reduced GFR	GI ulceration, thrombosis, rash	314, 319, 320
disulfiram	2E1 > 1A2	2E1	metallic taste, fatigue, *	hepatitis	139, 312, 323
itraconazole	3A4	3A4	diarrhea, dizziness, rash, hypokalemia, LFT elevation	hepatitis	311, 317
ritonavir	3A4>2D6	3A4, 2D6	nausea, paresthesia, elevated cholesterol, LFT elevation	lipodystrophy	209, 311, 313, 314, 315
sertraline	2D6 [weak]	demethylation	diarrhea, fatigue, nausea, decreased libido	mania induction	318, 321

Discussed below are the nine drugs of CUSP9* with an updated brief review of data on the 6 retained drugs with a fuller exposition of data on two new substitute drugs itraconazole and ritonavir and one new drug, celecoxib. See the original CUSP9 paper [1] for more detailed drug reviews and discussion of thinking behind CUSP.

#### Aprepitant

Aprepitant is a 534 Da oral inhibitor of signaling at neurokinin-1 receptors (NK-1) for which 11 amino acid, 1.4 kDa Substance P is the natural ligand [9, 10]. Aprepitant is approved by the FDA, EMA, and widely used as treatment for cancer chemotherapy induced nausea and vomiting for which it is quite effective [9, 10]. It is remarkably free of side effects of its own. Since Substance P signaling at NK-1 is a growth stimulating element in many cancers [11, 12] aprepitant has been suggested as a treatment adjunct for these cancers [13, 14], including glioblastoma [15, 16].

*In vitro* apoptosis induction by aprepitant and growth stimulation by exogenous Substance P can be demonstrated in other cancers as well [17, 18, 19] but heroic doses well beyond those used in humans seem to be required to demonstrate *in vivo* tumor suppression. We optimistically interpret this as an example of “Nile Distributary Problem” where other growth stimulating pathways takeover for one that is blocked.

#### Artesunate

Artesunate is a 384 Da phytoderived drug commonly used worldwide in treatment of malaria [20]. It is one form of the many related drugs derived from the same plant, collectively termed “artemisinins”. Artesunate converts rapidly after ingestion to its active metabolite dihydroartemisinin. Artesunate has inhibitory effects against, and is used in the treatment of various viruses, protozoa, helminths and fungi [21, 22]. Artesunate is particularly active against cytomegalovirus that are resistant to DNA polymerase inhibitors like valganciclovir [23, 24]. Artesunate has demonstrated empirical cytotoxicity against a variety of cancer cells [general review- 22, specific examples: lymphoma and myeloma, 25; pancreatic, 26; hepatocellular, 27; osteosarcoma, 28; gastric, 29; leukemia, 30, 31; glioma, 32].

In malaria studies artesunate is not given alone so its side effect profile is difficult to judge but it seem to be favorable. In short term studies of single agent artesunate 8 mg/kg/day in normals, altered taste and slight decrease in reticulocyte count were the only side effects noted [33].

Although clearly embryotoxic, and genotoxic [34] artesunate behaves clinically differently from traditional cancer chemotoxic agents. Mucositis, nausea, vomiting, bone marrow suppression, hair follicle synchronization typical of genotoxic chemotherapeutic drugs are not features of artesunate.

“If a little force doesn't work, maybe more force will”. Another of the principles in designing CUSP9 and its first revision CUSP9*, has been “ganging up”, hitting the same system at different points to block, or in the case of ROS increase, the given target mode of glioma cell death. Exposure of epithelial ovarian cancer cells to two of the CUSP drugs, disulfiram and auranofin, increased intracellular reactive oxygen species (ROS) mediated cell death to a greater degree than with either alone, as discussed below in their respective sections [35]. We add artesunate to increase ROS and cell death even further [36-38].

Also built on our ganging up directive was the combination of artesunate with a fourth CUSP9* drug, captopril, in order to inhibit neo-angiogenesis to a greater degree than by treatment with either drug alone [39].

Another area where we use the principle of ganging up is autophagy. Artesunate kills plasmodia and breast cancer cell lines via an autophagic pathway [40]. Artesunate decreased irradiation dose LD50 in LN229 and U87 glioma cell lines [41]. Of central importance to artesunate's use in CUSP9*, empirically artesunate augments temozolomide cytotoxicity in both U87 and A172 glioma cell lines [32].

Artesunate had good cytotoxicity against a panel of 91 cancer cell lines at *in vitro* levels well below the artesunate levels in patients treated for malaria [42]. In artesunate mediated plasmodia cytotoxicity, mitochondrial depolarization mediated by an increase in intracellular ROS was identified as mode of action [37, 38].

Artesunate lowered expression of thioredoxin and cytochrome c oxidase mRNA by 95% in schistosomes of infected mice [43]. A fifth CUSP9* drug auranofin is also a strong inhibitor of thioredoxin reductase, increasing intracellular ROS as discussed below.

#### Auranofin

Auranofin is a 679 Da gold-containing lipophilic drug first marketed in the 1980's for treatment of rheumatoid arthritis [44]. It remains in use worldwide as one of the so-called DMARDs, disease modifying anti-rheumatic drugs.

#### Auranofin and thioredoxin reductase

Thioredoxin reductase inhibition results in increased intracellular ROS. The effects of thioredoxin reductase inhibition by auranofin, the primary mode of action in both anti-rheumatoid arthritis and anti-cancer roles [45-48], can be significantly augmented by combination with disulfiram [35], another CUSP9* drug. Auranofin inhibited interleukin-6 induced STAT3 phosphorylation and nuclear translocation via an increased ROS step [49]. Chronic lymphocytic leukemia cells are similarly preferentially killed by auranofin generated ROS [50]. Like auranofin, other structurally unrelated inhibitors of thioredoxin reductase are cytotoxic to cancer cell lines by ROS generation [51-54].

An interesting feedback system [55] reminiscent in principle to that of Schruefer et al [vide infra, 56] and with potentially quite important clinical implications has been delineated since CUSP9 was published. Cell-free medium from glioma cell cultures stimulated synthesis of inducible nitric oxide synthase (iNOS) and consequent increased NO production in non-malignant microglia *in vitro* but without changing these normal microglia's proliferation or death rate [55]. This normal microglia produced NO in turn stimulated co-cultured glioma cells' migration and their synthesis of tumor necrosis factor-alpha (TNF-alpha) and macrophage chemotactic protein-1 (synonymous with CCL2, MCP-1) [55]. MCP-1 has an established growth promoting and negative prognostic effect in glioblastoma [57-59].

#### Auranofin and 5-lipoxygenase

CUSP9* has been modified from the original CUSP9 to account for a specific reciprocal back-and-forth shunting between cyclooxygenase (COX) and 5-lipoxygenase (5-LO). As one is inhibited, the other tends to become augmented in reciprocal fashion. This is discussed later in Section 2.6. on celecoxib and in Section 4. on reciprocal 5-LO and COX shunting.

Auranofin inhibited 5-LO in stimulated neutrophils [60-62] and in stimulated alveolar macrophages [63]. LTB4 is a major pro-inflammatory leukotriene product of arachidonate metabolism by 5-LO. Activated neutrophils' migration along an LTB4 gradient is also inhibited by auranofin [61, 64]. Neutrophil aggregation, degranulation, chemotaxis toward LBT4 as well as LBT4 synthesis itself was blocked by auranofin [65-67].

An early study gave evidence of auranofin's ability to cause arachidonic acid release from macrophages [63]. This was accompanied by some increase in thromboxane and prostaglandin E2, PGE2 [63]. Hence, for this reason also, to limit potential compensatory increase in PGE2, we add celecoxib to CUSP9* as discussed below.

#### Auranofin and cathepsin B

Cathepsin B is rather prominently upregulated in glioblastoma [16, 68-70] and promotes glioblastoma growth by contributing to matrix dissolution, Bcl-2 maintenance, and AKT activation [16, 68-70]. Cathepsin B becomes upregulated in a CXCL12 mediated feedback system with vessel endothelium [71] forming yet another positive mutually reinforcing feedback system active in glioblastoma with negative consequences in that glioblastomas are prodigious synthesizers of CXCL12, a target of treatment in its own right [72, 73]. A drawback is that auranofin is only a weak inhibitor of cathepsin B activity [16, 74]. But it is the only one we've got.

#### Captopril

Captopril is 217 Da angiotensin converting enzyme, ACE, inhibitor [75]. It was the first marketed pharmaceutical ACE inhibitor and has remained in wide use since its clinical introduction in the early 1980's. Captopril and a dozen other marketed ACE inhibitors are supported by a robust database on clinical effectiveness in treating hypertension, chronic heart failure, and as renoprotection during glomerulonephropathies [76]. Although more convenient once-a-day ACE inhibitors have since come on the market, we chose to use captopril because so much of the empirical anti-cancer data was collected specifically on captopril. Although we probably have a class effect, we cannot safely assume this.

#### Captopril as an anti-cancer agent

We include captopril in CUSP9* based on three not necessarily discrete lines of evidence: a] data showing that captopril inhibits activity of soluble matrix metalloproteinase (MMP) -2 and MMP-9, with a parallel data set showing these MMPs to be a growth facilitating factor in glioblastoma growth, b] ACE inhibitors, including specifically captopril have been shown to inhibit angiogenesis, both normal and cancer-related, c] empirical studies showing growth inhibition in cancer models. These will be reviewed with references below.

As an anti-glioma agent, captopril was first mentioned in 1995 when exposure *in vitro* resulted in diminished invasion, growth and MMP-2/MMP-9 activity [77]. These results were later replicated [78, 79]. MMP-2 and -9 inhibitory properties of captopril were reviewed in 2012 [80]. Many cancers have been shown to over express MMP-2, -9. The principle of our use of captopril in glioblastoma was active in a study of renal cell carcinoma (RCC), where immunohistochemical evidence for ACE expression on RCC, was first demonstrated in 1983 and this ACE activity was inhibited by captopril [81].

Throughout mammalian physiology, we see the pattern where different organs or organ systems use the same or similar function-mediating system for different ends, ends specific and relevant to the differing goals served by the specific organ or organ system. The renin-angiotensin system (RAS) is one such system. While most commonly thought of in terms of mediating the organ cross-talk between kidney-liver-lung in regulating sodium chloride balance and blood pressure, all components of RAS are endogenously expressed and used within brain and bone for examples, as semi-autonomous systems. Semi-autonomous means that both fully autonomous within bone or brain and elements interacting with systemic RAS are recognized.

Of special importance to CUSP9*, Carpentier et al demonstrated a steroid-sparing effect of ACE inhibitors or the related class of drugs, angiotensin receptor blockers, ARBs in glioblastoma patients [82]. We discuss their findings further below, offering the interpretation that glioblastoma overexpressed ACE functions to enhance peritumoral edema.

In a clinical study of low dose captopril, 12.5 mg twice daily, post-resection PSA rises occurred in 3 of 32 prostate cancer patients in the captopril group versus 10 of 30 in the control group [83]. Crucially, follow up data on OS have not been published. Others are looking at slowing or stopping cancer growth by blocking growth factors with repurposed already-approved drugs. In 2004, Jones et al studied three non-cytotoxic drugs- captopril 50 mg twice daily in combination with marimastat and subcutaneous fragmin- in patients with various terminal metastatic cancers [84]. Whether this treatment regimen slowed disease progress as the authors claimed or not, the drugs were well-tolerated even in this very sick patient population [84].

Marginal reduction in prostate cancer (relative risk was o.7) in those using captopril in hypertension treatment was seen in an epidemiological study of 23,000 men that showed no association- increased or decreased- relative risk in users of any other antihypertensive drug suggesting a captopril-specific rather than class-specific effect [85]. In rat prostate cancer, Wilson et al demonstrated captopril inhibitable ACE expression, conjecturing that ACE matures multiple growth factors by proteolysis of the parent peptide [86], a conjecture with which we agree.

Captopril inhibited tumor growth in a murine colon cancer model [87]. Growth of a human gastric cancer cell line [88] and a myelogenous leukemia cell line [89] were inhibited by low dose captopril. ACE and angiotensin II receptor 1, ATR1 were overexpressed in 19 of 25 resected pancreatic ductal cancers examined by immunohistochemistry and captopril suppressed their proliferation *in vitro* [90]. Captopril inhibited a renal cell carcinoma cell line's growth in severe combined immunodeficient mice without showing *in vitro* proliferation inhibition to that cell line at an equivalent concentration [91].

Estrogen receptor expression and *in vitro* proliferation of mammary ductal cell carcinoma cell lines were inhibited by captopril [92, 93]. After corneal injury neovascularization at injury site interferes with vision. Captopril inhibited this destructive post-injury corneal vascularization that was tractable to diminished endothelial cell migration at clinical captopril levels <10 micromoles [94]. Building on this observation, Volpert et al examined effects of captopril on a rat fibrosarcoma cell line that was resistant to captopril *in vitro*. Interestingly, they found *in vivo* captopril did inhibit this tumor's growth, suggesting an effect of stroma/host vasculature response rather than the malignant clone itself [94].

Captopril lengthened overall survival in mice with Lewis lung carcinoma and potentiated cyclophosphamide in this model [95]. Very high doses of captopril [~^50^ mg/kg/day] in mice inhibited fibrosarcoma and squamous cell carcinoma development in irradiated skin [96]. A xenografted human melanoma cell line's growth was inhibited by captopril [97]. However, and instructively, captopril had no antiproliferative effect on a variety of other cancer cell lines *in vitro* [98, 99]. Instructive in that clinical cancer growth inhibition via ACE inhibition occurs largely, although not exclusively, through angiogenesis inhibition, not inherent cytotoxicity.

Captopril inhibited the activity of soluble MMP-2 and MMP-9 secreted by a gastric adenocarcinoma cell line [100]. Growth in nude mice of this cell line was inhibited by captopril and inhibited yet further in combination with cisplatin [100.]. Captopril inhibited a mammary ductal cell carcinoma cell line in a Cu++ dependent manner, with indicators this was mediated by increased intracellular ROS [93]. Captopril inhibited soluble MMP-2 and MMP-9 activity from Lewis lung cancer cells *in vitro* and slowed growth in a murine model [101]. Moreover, in three triple-negative breast cancer cell lines tested, captopril inhibited proliferation only in those cells that expressed outer cell membrane ACE [102]. Importantly for CUSP9*, glioma cells express ACE-like activity [103].

Growth of murine colon cancer metastases to liver [104] and a xenografted non-small cell lung cancer cell line [105] were inhibited by captopril. Of particular importance and interest to the CUSP9* protocol, another CUSP9* drug, artesunate, when added to captopril yielded a synergistic inhibition of angiogenesis in an ovine allantoic membrane angiogenesis model [39].

#### Captopril and tissue factor

Tissue factor (TF, factor III, also termed thromboplastin), is a 47 kDa outer cell surface receptor for soluble, activated clotting factor VII [106.]. The TF-factor VII complex then mediates factor X conversion to activated factor Xa. TF is commonly elevated in human glioblastoma [107-110]. TF is yet another of the many growth facilitating factors we aim to inhibit in CUSP9*. In a breast cancer cell line, TF activity and TF mRNA was reduced by about a third by captopril exposure *in vitro* [111]. TF subserves other growth enhancing aspects of glioblastoma growth in addition to contributing to the excess thrombosis related morbidity associated with glioblastoma [106, 108, 110, 112-115]. This is so particularly in the stem cell subpopulation [114, 115].

The dangers of systemic anticoagulation in glioblastoma are clear [111, 116]. Captopril mediated down-regulation of glioblastoma cell overexpression of TF might be an ideal way to deprive glioblastomas of this growth and migration enhancing system.

### Celecoxib

#### Introduction

New to CUSP9* is celecoxib. Celecoxib is a 381 Da cyclooxygenase (COX) inhibitor commonly and effectively used to treat pain of diverse origins [117, 118]. It lacks any platelet aggregation inhibitory activity as seen with some other COX inhibitors [119]. Clinical and research literature routinely call celecoxib “COX-2 selective” and this is relatively so but to our reading of the data celecoxib inhibits COX-1 to some degree [120-123 vide infra]. Shortly after clinical introduction to clinical practice in symptomatic treatment of pain, anti-cancer effects were noted both empirically and by theoretical reasoning [124]. Celecoxib is now being widely used on and off cancer treatment protocols in a variety of different cancers. It has come to our attention that people with glioblastoma are commonly starting celecoxib on their own, often without their oncologist's knowledge.

#### Celecoxib and COX-1, COX-2

Celecoxib is currently in 42 open clinical trials as adjuvant to traditional cancer cytotoxic or drugs or treatments [clinicaltrials.gov]. Celecoxib adjuvant trials reporting recently have not been entirely negative but neither have they been strongly encouraging. Many were uncontrolled and had other variables that didn't permit benefit quantitation. Adjuvant celecoxib was generally well tolerated in these studies.

In an uncontrolled study, children with various advanced cancers were given cyclophosphamide and etoposide as cytotoxic chemotherapy augmented with non-cytotoxic growth factor blocking drugs [thinking along same lines as CUSP9*] celecoxib, thalidomide, and fenofibrate [125]. The authors, experienced pediatric oncologists, considered “Clinical activity was demonstrated in some but not all tumor strata.” An uncontrolled study in advanced metastatic breast cancer of daily cyclophosphamide plus celecoxib 200 mg twice daily “is safe and shows a therapeutic effect in advanced breast cancer patients” [126]. In another study, a treatment regimen consisting of celecoxib 200 mg twice daily, 5-fluorouracil, epirubicin and cyclophosphamide followed by docetaxel was reported to be “active and safe for treatment of operable invasive breast cancer” [127]. In addition, in a study of carboplatin, gemcitabine or carboplatin with vinorelbine in advanced non-small cell lung cancer given celecoxib at 400 mg twice daily “The effect on survival by celecoxib in the whole subset of patients was positive” [128].

However, there are also negative studies. For example, a study of advanced epithelial ovarian cancer treated with docetaxel, carboplatin using adjuvant celecoxib at 400 mg twice daily showed no survival benefit from added celecoxib [129]. We refer to the reciprocal relationship between COX and 5-LO, discussed in detail with references in section 4. below as explanation for this failure and how we address this in CUSP9*.

#### Celecoxib and carbonic anhydrase

Carbonic anhydrase catalyses the reaction CO2 and H2O to H+ and bicarbonate. It has 12 isoforms of which some are soluble in the cytoplasm [isoform -2], some transmembraneous [isoforms -9 and -12]. In either case, the proton is excreted and bicarbonate kept within the cell. The net effect is further acidification of the extracellular milieu. Glioblastoma cell carbonic anhydrase thereby contributes to glioblastoma's acidic milieu [vide infra]. In 2006, a blind selection of 960 molecules tested for carbonic anhydrase-2 inhibition revealed that celecoxib was among the most potent molecules [130].

Glioblastomas, like many other cancers, express abnormally large amounts of carbonic anhydrase [131-134]. Stronger carbonic anhydrase-9 immunohistochemical staining of human glioblastoma biopsies was correlated with shorter overall survival, a robust finding confirmed by four independent groups [135-138]. In biopsy specimens of endothelium derived from glioblatomas, overexpression of carbonic anhydrase isoform-2 was found and likewise correlated with shorter survival [135]. Overexpression of carbonic anhydrase is thought to be an adaptation allowing better survival in the hypoxic, acidic environment of tumors [134, 135], particularly glioblastomas [138]. Although areas of most intense immunohistochemical staining for carbonic anhydrase tend to be the most hypoxic, and or necrotic, it seems that in fact it is extracellular acidosis that triggers increased expression of carbonic anhydrase [131, 137].

The pan-carbonic anhydrase inhibitor acetazolamide was shown to enhance temozolomide cytotoxicity in a glioma cell line while dexamethasone reduced temozolomide cytotoxicity in this same model [138]. Writing in 2008 these authors suggested clinical use of acetazolamide along with temozolomide to reduce peritumoral edema, enhance temozolomide's cytotoxicity, and reduce need for [potentially counter-productive] use of dexamethasone. We agree and choose celecoxib partly for its nanomolar inhibition of carbonic anhydrase.

#### Disulfiram

Disulfiram is a 297 Da drug, used since the 1950's to treat alcoholism by making ethanol ingestion highly unpleasant [1, 139-142]. As a potent inhibitor of all isoforms of aldehyde dehydrogenase, ALDH, disulfiram stops ethanol metabolism at the acetaldehyde stage. Multi-system dysfunction ensues if ethanol is consumed, as manifest by flushing, hypotension, malaise, nausea, vomiting. Acetaldehyde is poorly tolerated. ALDH normally would transform the toxic ethanol metabolite acetaldehyde into non-toxic acetic acid that is smoothly handled without problem. Since disulfiram chelates Cu++ in the stomach even without adding exogenous Cu [139], Cu gluconate of CUSP9 has been deleted in CUSP9*.

In CUSP9 [1] we gave the basic rationale for adding disulfiram to glioblastoma treatment. Since then the rationale has been further elaborated and details added [35, 143, 144]. We give here in CUSP9* a short background and a little update on disulfiram developments since end of 2012.

The original suggestion to use disulfiram in treatment of glioblastoma came from dozens of reports associating high ALDH expression in individual cells with those cells having stem cell attributes [142, 143]. This held true in various cancers including glioblastoma [141, 142, 146]. There are currently 30 open studies on disulfiram as adjuvant treatment in various cancers [clinicaltrials.gov].

Some recent data on disulfiram: Mesothelioma cells' growth inhibition by disulfiram/copper was associated with weaker NFkB activation, accumulation of ubiquitinated proteins and cleavage of vimentin [145]. A study in glioblastoma also showed disulfiram cytotoxicity was related to inhibition of NFkB activation [146- 148]. Cytotoxicity by disulfiram/copper to non-small cell lung cancer cells resulted in cell cycle arrest at G2/M [147]. In hepatocellular cancer disulfiram/copper mediated cytotoxicity was shown to be mediated through an ROS increasing step [148], findings concordant with our findings on disulfiram/copper cytotoxicity to epithelial ovarian cancer cells being mediated in part by increased ROS [35, 149]. Likewise, pancreatic cancer cell growth inhibition in a murine xenotransplant model was also ROS dependent [150]. A compound screen including several thousand drugs showed prostate cancer cells to be sensitive to disulfiram which was partially reversible by adding ROS scavenging agents [151] again indicating that disulfiram-mediated cytotoxicity at least in part is due to the increased generation of ROS. ROS increase as a primary mode of disulfiram-mediated cell death was already indicated in early works with melanoma cells over ten years ago [152, 153].

In the course of replicating disulfiram's increased ROS cytotoxicity to breast and colon cancer cells, and quite happily for our intended use, hypoxia and lower pH – conditions prevailing within glioblastoma tissue - both enhance disulfiram's cytotoxicity [154]. Disulfiram alone, but particularly so in combination with auranofin, results in large increases in intracellular ROS [35].

In a genomic study examining a panel of ovarian cancer cells ALDH expression as a feature of stemness was again confirmed suggesting disulfiram as a stemness defeating drug for that type of cancer [155]. As for other cancers, the subset of high ALDH expressing cells was associated with stem cell attributes and disulfiram again, as predicted, defeated much of the stem cell functions, both *in vitro* and in a xenograft model [156]. Breast cancer cells expressing ALDH were shown to have decreased sensitivity to paclitaxel and cisplatin compared to ALDH non-expressing cells. Decreased sensitivity in these ALDH expressing cells reverses after exposure to disulfiram [157]. Triple negative breast cancer cells showed synergy in cytotoxicity when disulfiram is added to doxorubicin [158]. Gemcitabine cytotoxicity is enhanced by disulfiram in glioblastoma cells [159], in pancreatic cancer cells [156, 160] and in breast and colon cancer cells [161].

However, disulfiram related cytotoxicity seems to be exerted by at least two main paths- one Cu++ dependent, one not [35, 162, 163]. Importantly, for our intended use cancer cell specific cytotoxicity peaks when disulfiram and copper are present in a 1:1 molar ratio [162]. By an unclear mechanism, cytotoxicity is reduced by excess disulfiram [162]. Data from early 2014 indicate that disulfiram is a partial inhibitor of the main temozolomide damage repair enzyme, O6-methylguanine-DNA methyltransferase, MGMT [163].

However, a study of disulfiram 250 mg/day or 500 mg/day as isolated treatment failed to significantly affect progression in men with localized recurrent post-resection prostate cancer as measured by PSA slope [164]. We interpret the discrepancy with *in vitro* data to mean a] in human disease there are compensatory paths engaged when ALDH is inhibited, and b] these compensatory growth paths must also be blocked simultaneously with ALDH. Certainly, there are also other interpretations for the failure of disulfiram to change prostate cancer growth slope.

It still remains a matter of debate whether disulfiram's anti-cancer mechanism of action is based on ALDH inhibition, MGMT inhibition, NFkB activation inhibition, proteasome inhibition, or increased intracellular ROS generation. Is one event primary and the others secondary to that one? Or is stem cell selectivity achieved by a combination of actions? Answers to these important questions are currently unknown.

### Itraconazole

#### General

Itraconazole is a 706 Da broad spectrum anti-fungal agent used clinically since the late 1980's [165, 166]. It is commonly used today for onychomycosis and as anti-fungal prophylaxis, or as an empirical treatment for fever, in neutropenic patients [167]. As with many other highly lipophilic drugs, brain tissue concentration is greater than CSF [165]. Although 5% of itraconazole treated patients develop some elevation of LFTs, this has lead to overt liver damage in < o.o2% of treated patients [165].

In a study initiated on the basis of preclinical evidence for itraconazole's inhibition of angiogenesis, Rudin et al recently showed prominent lengthening of OS in itraconazole plus pemetrexed treated metastatic nonsquamous non-small cell lung cancer patients when compared to those receiving pemetrexed alone [168]. Based on positive murine prostate cancer xenograft studies, Antonarakis et al in a clinical study found clear evidence for itraconazole's anti-tumor effect- reduced PSA levels [of note without effect on androgen levels], shallower PSA slope, and fewer circulating cancer cells in metastatic prostate cancer patient [169]. Skin biopsies derived from these patients showed diminished Hedgehog, Hh, signaling, [vide infra], a finding concordant with studies on mesothelioma cells exposed to itraconazole [170] and murine Hh signaling studies with itraconazole [171].

Proton pump inhibitors, H2 inhibitors, and other stomach acid reducing agents must be avoided during itraconazole due to decreased absorption at higher pH [172]. In HIV patients receiving ritonavir 400 mg twice daily, the related azole antifungal drug ketoconazole increased cerebrospinal fluid ritonavir (from 2.4 to 6.6 ng/mL) to disproportionately greater degree than it raised blood ritonavir levels [173]. This was a major reason for adding ketoconazole to the original CUSP9 regimen [1]. We believe itraconazole will achieve similar increased ritonavir concentrations based on itraconazole's similar efflux pump inhibition as that seen with ketoconazole [174, 175].

From the outset of our work constructing a non-cytotoxic adjuvant protocol for cytotoxic treatment of recurrent glioblastoma we considered 5-LO inhibition as an important component. In a screen of hundreds of FDA approved drugs across multiple classes, none of which were traditional cancer chemotherapeutic drugs, Chong et al found itraconazole to be a specific inhibitor of human umbilical cord endothelial cell proliferation while surprisingly having little or weak inhibitory effect on normal fibroblasts, Jurkat T cells or HeLa cells [176]. In support of our substitution of ketoconazole in CUSP9 with itraconazole in CUSP9*, ketoconazole was shown to provide less anti-angiogenesis activity in this model. Chong et al's premise was to “uncover novel biological activity among existing drugs,” [176] - precisely one of our main driving principles also in CUSP9*. Another group showed diminished nascent capillary sprouting as well as endothelial proliferation inhibition during exposure to itraconazole [177].

Easily achievable levels of itraconazole will block vascular endothelial growth factor, VEGF, binding to VEGF receptor-2 [178]. This was traced to defective receptor trafficking, which in turn was secondary to itraconazole mediated defective glycosylation of VEGF receptor-2 [178]. Another interesting and helpful aspect of this trafficking inhibition by itraconazole is its interference with mTOR activation [179].

mTOR/AKT signaling in glioblastoma cells was inhibited during exposure to itraconazole with autophagic cell death to follow [180]. Since “angiogenesis is dependent on multiple growth factors and a broad signaling network *in vivo*” [180] it seems reasonable that we must block multiple growth factors with multiple drugs, until and unless we can find a crucial single sine qua non element in this process. So far, it seems there is none such. Hence, at least for now, CUSP9*.

The pharmacokinetics of itraconazole oral solution was measured in seven patients receiving chemotherapy followed by autologous bone marrow transplantation for leukaemia or lymphoma. Patients receiving itraconazole 5 mg/kg/day [70 kg = 350 mg/day] gave pre-dose serum level of ~800 ng/mL at steady state [about two weeks] [181]. A retrospective analysis of patients with acute lymphoblastic and acute myelogenous leukemia receiving daunorubicin where itraconazole was used as antifungal prophylaxis during the neutropenic nadir showed better remission rates than those not receiving itraconazole [182]. The effect was not large but was statistically significant. *In vitro*, intracellular levels of daunorubicin increased proportionately as itraconazole levels went from o.5 to 5.0 microg/ml [183].

#### Special position of 5-lipoxygenase

We now place greater emphasis on 5-LO in CUSP9* than we did in CUSP9. Leukotrienes are arachidonate derived signaling molecules of importance that are overexpressed in many cancers [184] and demonstrated to be so specifically in glioblastoma [185-189]. Given that glioblastomas express excess 5-LO and that 5-LO product leukotrienes are major mediators of glioblastoma-related brain edema [185] we predict therefore that itraconazole will lower brain edema and the consequent need for dexamethasone. Steroid-sparing action alone would be expected to improve QOL and OS [82, 190].

Itraconazole at low micromolar concentrations inhibits synthesis of leukotriene LTB4 in activated neutrophils even more deeply than does ketoconazole or miconazole [191-193]. “Changes across metabolic networks” [194] that are not malignant by themselves become an integral part of malignant growth when combined with the suite of so deranged networks and genomic changes driving them. We aim to dismantle or block enough of these networks to hobble glioblastoma growth. Morin et al thinking along these lines found that zileuton, a specific drug marketed as an 5-LO inhibitor [to treat allergy symptoms] interferes with glioblastoma cell line growth [194] as previously hypothesized by Omahen in 2011 [195]. Herbal-derived 5-LO inhibitors [“Nordy” and others] have also shown anti-glioma growth activity, activity in xenograft model, cytotoxicity that exceeded that of the classical alkylating cytotoxic drug carmustine (BCNU) [196]. Itraconazole blocks 5-LO activity without affecting activity of COX *in vitro* in low micromolar concentrations [192], levels easily achieved in clinical use.

Much of our aim in CUSP9* is inhibiting or blocking normal cell processes pathologically employed by cancer cells to grow or evade senescence, apoptosis or cytotoxicity. Others are thinking along these lines also [196, 194, 196, 197].

Some glioblastoma patients currently use cannabidiol, based on preclinical studies [198-200], internet chatroom discussions, and anecdotal reports of benefit. In a formal study of cannabidiol, much of the *in vitro* anti-cancer and specifically anti-glioma effects derive from its 5-LO inhibition. Cannabidiol is one of the many biologically active but not psychoactive molecules present in marijuana. It is sold openly, legally, and without prescription in most jurisdictions on the herbal market.

Again within the principle of ¨ganging up¨ disulfiram as well inhibited rat neutrophil soluble 5-LO with IC50 <1 micromol [201]. Diethyldithiocarbamate, the prominent circulating metabolite of disulfiram, still had 5-LO inhibiting properties but was less potent than its parent compund [201]. Concordant with that work, disulfiram inhibited LTB4 release from isolated human neutrophils at IC50 <5 micromol and in an *in vivo* rat model but less effectively so [202]. Thinking along these lines of “ganging up”, similar to our CUSP9 protocol, Jiang et al reported earlier this year [196] results from a drug screen empirically looking at a thousand non-cytotoxic, non-cancer chemotherapy-related FDA approved drugs for cytotoxicity against glioma cell lines [196]. Two of the CUSP9* drugs, itraconazole and sertraline happened to have- as predicted in CUSP9 last year- to have good anti-glioma cell activity (at least *in vitro*) [196].

#### Itraconazole and p-gp

Itraconazole significantly inhibited breast cancer resistance protein, BCRP, efflux pump, thereby lowering cytotoxicity resistance to topotecan [203] and leukemia cell line resistance to doxorubicin and etoposide [204].

P-glycoprotein [p-gp] is a 170 kDa, ATP consuming, outer cell membrane drug [xenobiotic molecule]efflux pump synonomous with MDR-1 [205, 206]. It preferentially exports lipophilic molecules, a veritable intracellular “hydrophobic vacuum cleaner” [205] particularly active at the blood-brain barrier (BBB) endothelium [206]. Itraconazole is both an efflux substrate and partial inhibitor of p-gp [206-208].

Recognizing itraconazole's remarkably useful attributes of both Hh signaling inhibition and p-gp inhibition, a retrospective chart review showed that clear cell ovarian cancer patients receiving itraconazole 2 days before and during treatment with platinum and taxane had over twice the overall survival compared to those receiving the same chemotherapy regimen but without itraconazole and had even greater effect in prolonging survival in other forms of epithelial ovarian cancer [208].

#### Ritonavir

Ritonavir is a 721 Da protease inhibitor, the first such approved for use in humans to treat HIV [209]. As mentioned above, ketaconazole more than doubles CSF ritonavir levels (2.4 to 6.6 ng/mL) in HIV positive people [173]. Concomitant administration of ritonavir (400 mg twice daily) plus ketoconazole [200 mg twice daily] was well tolerated and resulted a in disproportionate increase in CSF ritonavir level compared to a small increase in plasma level [173]. Both drugs, ritonavir and ketoconazole, as well as itraconazole, are substrates for and inhibitors of p-gp and MRP1.

A trial of ritonavir 100mg with lopinavir 400 mg twice daily reporting in 2011 showed little or no benefit in prolonging OS of recurrent glioblastoma [210]. Of uncertain significance, there was one complete radiological remission that lasted 11 months before progression [210]. Parenthetically, note that in this report the published doses of ritonavir and lopinavir were reversed from the doses actually given. The authors have assured us that he standard commercial capsules of Klatra^TM^ (ritonavir 100mg with lopinavir 400 mg) were used.

Ritonavir inhibited *in vitro* proliferation of a glioma cell line [211]. Ritonavir *in vitro* exposure increased apoptosis and decreased proliferation of pancreatic ductal adenocarcinoma cell lines which led the authors to conclude that using ritonavir in pancreatic duct cell cancer by “drug repositioning…would limit the costs and reduce risks” [212]. Our point exactly times nine.

Sato et al demonstrated renal cell carcinoma proliferation inhibition by ritonavir in 2012 [213]. Cervical carcinoma in situ cells synthesize both MMP-2 and MMP-9. Activity of both was reduced by ritonavir with corresponding *in vitro* invasion inhibition [214]. Non-small cell lung cancer cell lines' growth was inhibited by ritonavir as well [215].

The eleven members of the ABC efflux pump group are associated with cancer chemotherapeutic drug efflux from cells and therefore become an element of chemotherapy resistance. Breast cancer resistance protein (BCRP), p-gp, or various multidrug resistance proteins are better known members of this group and all are inhibited to varying degrees by ritonavir [216]. Kumar et al showed that ritonavir at 20 microM generated G1 cell cycle arrest and apoptosis in ovarian cancer cell lines MDH-2774 and SKOV-3 [217]. Kumar et al conclude that HIV protease inhibitors like ritonavir “are efficient blockers of MDR1 (P-gp), MRP1 and BCRP” [217]. Ritonavir induced cell-cycle arrest at G1-phase and apoptosis in EBV-positive lymphoblastoid B cells *in vitro* [218].

Decreased AKT phosphorylation is a basic mode of action in ritonavir cytotoxicity [212, 217, 219]. Again the refrain, “[ritonavir] repositioning for ovarian cancer… would reduce risks, limit the costs and decrease the time needed to bring the drug from bench to bedside” [219]. We concur.

Ritonavir increases etoposide cytotoxicity in MRP-1 over-expressing cells [220]. Long-term exposure to ritonavir seems to upregulate p-gp expression [221] but at the same time ritonavir inhibits the efflux of paclitaxel and vinblastine in p-gp-positive cell lines [222]. Ritonavir inhibited the xenobiotic export pump BCRP in low micromolar concentrations but was not a substrate for BCRP [223, 224]. A rat glioblastoma cell line's *in vitro* proliferation was suppressed by ritonavir, and direct inhibition of proteasomal chymotrypsin-like activity by ritonavir could be demonstrated. However, in an *in vivo* rat model ritonavir had no effect on glioma growth [225].

To what extent the many paths of ritonavir-mediated cell death in cancer cell lines and relative resistance to such death in non-malignant cell lines [226] is fundamentally secondary to AKT inhibition or not, is unknown.

Like itraconazole, ritonavir is both an efflux substrate and partial inhibitor of p-gp [227-229]. Ritonavir penetration into normal brain tissue is poor but as mentioned above adding ketoconazole increased cerebrospinal fluid concentrations of ritonavir (from 2.4 to 6.6 ng/mL) in those receiving 400 mg ritonavir twice daily [183]. We expect itraconazole to similarly assist ritonavir penetration of the BBB.

Several multiple myeloma cell lines responded to ritonavir with decreasing proliferation and increasing apoptosis [230]. Ritonavir is a strong inhibitor of hepatic P450 3A4 and a clear but relatively weak inhibitor of p-gp efflux pump, but together these two attributes ended up increasing orally administered circulating docetaxel 50 fold [231]. Human endothelium growing *in vitro* show mitochondrial DNA damage and reduced proliferation after exposure to ritonavir [232.]. Colon carcinoma cell apoptosis was increased after exposure to ritonavir [233]. Ritonavir is a strong hepatic P450 3A4 inhibitor that lowered affinity of activated NFkB with its DNA target sequence [233]. Ritonavir enhanced radiation-induced apoptosis in a murine model of head and neck squamous cell carcinoma [234].

### Sertraline

#### Introduction

Sertraline is a 306 Da antidepressant of the selective serotonin re-uptake (SSRI) class with a minimal side effect profile [235]. Sertraline is widely used worldwide. It is a mainstay in current treatment of excessive anxiety states as well. Side effects are usually well-tolerated consisting of reduced libido (occurs in one third of treated patients) and some loosening of bowel movements (in one tenth of those treated). Other side effects are rare [235]. Sertraline is also commonly used in metastatic cancer with co-morbid depression where it is a safe and effective antidepressant/anti-anxiety agent with side effects not different from that seen when used in non-cancer settings [236].

Sertraline was included in the original CUSP9 protocol partly based on the empirical observation that glioblastoma patients on SSRI class antidepressants had a longer OS than those not on SSRIs [237]. The authors of that study reported on a 1.6 month longer OS in those treated with SSRIs which was not statistically significant but we considered the possibility that if a larger cohort continued to show similarly longer OS this would become statistically significant [1]. The eminent safety and tolerability of sertraline made the risk/benefit skewed enough to include sertraline in CUSP9*.

#### Sertraline effects on cancer cells

Sertraline was shown to induce cytotoxicity in a human osteosarcoma cell line [238], a human prostate cancer cell line [239] and a squamous oral cancer cell line [240] by mediating phospholipase C -dependent Ca++ release from the endoplasmic reticulum, ER, and Ca++ cell entry by L-type Ca++ channels. Inhibition of proliferation by sertraline of colon cancer cell lines, both *in vitro* and when xenografted, was traced to Bcl-2 inhibition [241]. In a drug pair study of sertraline in five glioma cell lines (U87MG, U343MG, U373MG, A172, T98G) robust inhibitory activity was seen across these cell lines [242].

Translationally controlled tumor protein, TCTP, is an ancient ~20 kDa intracellular chaperone protein dysregulated in mammalian cancers [243]. TCTP is seemingly closely related to heat shock proteins [244]. Sertraline inhibits TCTP [245-247].

An important role of TCTP relating to glioblastoma's treatment-resistance is TCTP's non-covalent bonding to p-53 preventing p-53's function [248]. p-53 is a 44 kDa protein active in both normal and malignant cells that, although a multi-functional protein, i.a. is a transcription factor leading to G1-arrest and/or apoptosis during cytotoxic chemotherapy. Thereby p-53 becomes most important in cancer research and hence in CUSP9* [249]. In glioblastoma, TCTP overexpression destabilizes p-53, preventing p-53's pro-apoptosis signaling during chemotherapy [250]. By sertraline's inhibition of TCTP [245-247] we intend to restore some p-53 function.

Glioblastoma biopsy with TCTP overexpression indicates a more aggressive clinical trajectory, have a higher proliferation rate, and were associated with shorter OS [250, 251]. Stemming from observations that revertant subclones [sub-clones with reduced malignant characteristics]of cancer cell lines had reduced TCTP [247], sertraline was noted to both reduce TCTP and enhance the reversion process [247]. An inverse reciprocal quantitative relationship exists between TCTP and p-53. TCTP enhanced p-53 degradation while p-53 repressed TCTP transcription [245, 246]. The repressive function of TCTP is inhibited by sertraline in a breast cancer cell line, thereby de-repressing p-53, allowing resumption of p-53 mediated apoptosis [245].

Empirically, sertraline inhibited glioma cell line U87 proliferation at even lower concentrations than did temozolomide [252]. Proliferation inhibition at low micromolar *in vitro* concentrations was also seen in a breast cancer cell line that was traced to mTOR inhibition [253]. Jurkat cell line proliferation was inhibited by sertraline at lower concentration than by vincristine or cyclophosphamide and cytotoxicity of vincristine and doxorubicin was enhanced by clinically achievable sertraline levels [254]. Melanoma xenograft growth was inhibited likewise by sertraline at clinically achievable doses and correlated with inhibition of AKT phosphorylation[255].

The sertraline dose has been increased from 50 mg twice daily to 100 mg twice daily in CUSP9* based on early reports of good tolerability, increased fatigue has been the main side effect noted from the increase, in those who have taken it as part of compassionate use in CUSP9.

### Hedgehog signaling pathway

#### General

The hedgehog signaling pathway, Hh, [256] is one of the growth driving signaling systems in cancer generally [257, 258] and in glioblastoma specifically [259, 260]. Autocrine, juxtacrine and paracrine Hh activation modes are recognized. Hh signaling is a multi-stage process starting at the base of cells' cilia going to the cilia tip, then back to cells' nucleus. Glioblastoma cells bear cilia and they are faulty [261, 262]. Hh is a particularly important signaling system in that it branches out to transactivate numerous other signaling systems [257], particularly so and well documented in the case of glioblastoma [260, 263].

Vismodegib is a small molecule inhibitor of Hh that, by binding to SMO, tends to limit efficiency of Hh signaling [264, 265]. Vismodegib is FDA approved and marketed in USA/Canada, and several countries of the EU for treatment of metastatic or advanced basal cell carcinoma [265]. Basal cell carcinoma gives us an important lesson in understanding cancer in general and with specific reference principles that drove our CUSP9*.

In basal cell carcinoma, Hh plays a central role and acts in the absence of important enough cross-covering growth signaling pathways such that effective tumor growth suppression can be achieved with pharmacologic inhibition using the small molecule drug vismodegib [264, 265, 266], inhibiting a single pathway. An easy enemy to defeat.

Many other cancers where Hh has been shown to have important growth stimulating role cannot be so easily suppressed. In these cases, single agent inhibition of Hh is not efficient. The lesson: basal cell cancer is an indolent disease of low degree malignancy. Metastases are rare or a late occurrence in disease course. Bulk tumor invasion is clear but not very cancer-like (cf. cancer as crab). Simple surgical resection commonly results in cure. The microscopic invading tentacles common to other cancers and responsible for local recurrence and metastases are not usually seen in basal cell cancer. Corresponding to this it seems that simple blocking of a single signaling path - Hh - stops growth. The fact that vismodegib doesn't cure other cancers we take as evidence for multiple cross-covering growth signaling paths being generally active in cancers and specifically in glioblastoma. Hh has well documented partially cross-covering intersections with four other growth promoting pathways-1) RAS/RAF/MEK/ERK, 2) PI3K/AKT/mTOR, 3) EGFR [257]. Again we face the Nile Distributary Problem and aim to address it with a multi-drug regimen- CUSP9*.

#### Hh and Itraconazole

As mentioned itraconazole inhibits Hh signaling [170, 171]. Murine medulloblastoma growth inhibition by itraconazole was shown to act via Hh, specifically by blocking SMO translocation [171, 267]. In accord with previously documented Hh inhibition, a clinical study showed that itraconazole at 100 mg twice daily inhibited Hh signaling and proliferation in basal cell carcinomas [267]. Of particular interest, confirming our understanding of basal cell carcinoma, is the observation that basal cell carcinomas previously exposed and resistant to vismodegib showed no effect subsequent to itraconazole treatment [267]. If these cancers were resistant to one Hh inhibitor they were resistant to the other.

Mesothelioma cells were killed by Hh inhibition by itraconazole to an equivalent degree as did Hh RNA knockdown [170]. Itraconazole has significant anti-myeloma cell activity via Hh inhibition, particularly so in the ALDH co-expressing sub-population indicating Hh as a stem cell feature [268].

Based on strong *in vitro* and *in vivo* (xenograft) inhibition of endothelial cell proliferation, migration, and tubule formation in response to vascular endothelial growth factor (VEGF) and basic fibroblast growth factor in non-small cell cancer models [269], a phase II study compared standard cytotoxic therapy with or without daily oral itraconazole in recurrent metastatic non-small cell lung cancer [270]. The authors reported “trends suggestive of improved disease control” in the cohort treated with itraconazole 200mg/day [270].

In considering these data showing itraconazole as both p-gp efflux inhibitor and an Hh inhibitor, Tsubamoto et al found evidence for clinical activity in a 2014 clinical trial of itraconazole in advanced clear cell epithelial ovarian cancer [208].

High dose itraconazole [600 mg/day] given to men with advanced prostate cancer resulted in halving of the PSA level without changes in androgen [testosterone and dehydroepiandrosterone] levels [271]. Intriguingly and without current explanation, patients' aldosterone dropped to 25% of pre-itraconazole levels [271]. This latter finding is particularly felicitous in that a study from 1994 showed increased CSF aldosterone in brain tumor patients of diverse pathologies including glioblastoma, which was correlated to brain edema [272]. Since brain [273], and glioblastoma tissue itself have autonomous functioning renin-angiotensin systems [80, 274, 275], our expectation is that much of itraconazole's brain edema prevention derives from this diminished aldosterone synthesis during itraconazole treatment and will be augmented by our use of captopril.

### Reciprocal 5-LO and COX shunting

Recent findings of a reciprocal shunting between COX-2 and 5-LO [195, 276, 277] referred to earlier, has necessitated adding COX inhibition (with celecoxib in CUSP9*) along with the 5-LO inhibitor itraconazole. This would be then a specifically delineated case of addressing the Nile Distributary Problem. D.A. Omahen, noting increased expression of both COX-2 and 5-LO in gliomas, suggested in 2011 treating gliomas with simultaneous COX and 5-LO inhibitors ¨using readily available, well-tolerated medications¨, ¨thereby priming glioma cells for treatment-induced apoptotic cell death¨ [195].

Examples of COX/5-LO reciprocal relationship tendency: Smokers with elevated urinary PGE metabolite levels showed decreased urinary PGE metabolite levels after celecoxib administration but increased urinary levels of LTB4 [278]. Exposure of human chondrocytes to naproxen, a COX-1 and 2 inhibitor, increased LTB4 secretion and 5-LO mRNA [279].

Celecoxib had some 5-LO inhibition in a single *in vivo* study [280] but COX-2 inhibition predominated. Both COX-2 and 5-LO were up-regulated in head/neck squamous cancer cell lines [281]. COX-2 inhibition had little effect on proliferation but COX-2 inhibition did increase LTB4 [281]. Knockdown of both COX-2 and 5-LO gave significantly impaired proliferation and, parenthetically, VEGF production [281]. COX inhibitors suppressed PGE2 production but enhanced LTB4 secretion in COX-2 expressing colon cancer cell lines [278].

The reverse process can be seen as well. Some patients given the marketed pharmaceutical 5-LO inhibitor zileuton develop increased prostaglandin levels [282]. In a cardiomyocyte cell line zileuton upregulated COX-2 expression [283]. We have therefore potential for a bound bilateral see-saw [reciprocal] relationship between 5-LO and COX-2, with either cross-covering for the other. A specific example of the Nile Distributary Problem.

### Dosing and practical considations

#### Dose suggestions

Specific addition and uptitration schedules are available from the authors. The CUSP9* drugs are generally forgiving and have been well-tolerated in the handful of cases given CUSP9 drugs on a compassionate-use basis that have been reported to the authors to date. No remarkable toxicities have been reported. This benign side effect profile was expected based on wide experience over decades of use with these drugs.

Artesunate and auranofin are the only two drugs that might be unfamiliar to standard general medical practioners. Artesunate, although widely used to treat malaria around the world, is distinctly uncommonly used in developed countries due to the rarity of malaria there. Auranofin, although in use for several decades to treat rheumatoid arthritis, is uncommonly used. Captopril, celecoxib, ritonavir, and sertraline are common drugs, not rarely used together in day-to-day medical practice. Aprepitant is an unusually silent drug side effect wise when used in chemotherapy related nausea and vomiting control.

The doses for CUSP9* drugs are those commonly used doses when given for their approved indications- auranofin in rheumatoid arthritis, captopril for hypertension, celecoxib for joint pain, itraconazole for fungal infections. Doses for disulfiram and sertraline are slightly higher than average dosing in alcoholism or depression respectively, but CUSP9* doses below are at the higher end of the dose range given clinically. CUSP9* dose for artesunate, nominally ~ 1.4 mg/kg/day, is lower than that used in malaria treatment, ~ 4 mg/kg/day [284] but we will be using artesunate longer than is usual during malaria treatment and chose a conservative dose. Aprepitant is given at standard anti-emetic dose but for longer duration than is current clinical practice.

Weekly evaluations with complete blood count are essential given the potential for unforeseen drug-drug interactions in a medical regimen as complex as CUSP9*. Monthly MRI's will be done in the upcoming clinical trial. Both theoretical considerations [starting with the drugs least likely to give side effects [1]] and limited initial clinical experience suggest the following drug addition schedule: The initial four drugs and doses are aprepitant 80 mg once daily, auranofin 3 mg once daily, celecoxib 200 mg once daily, disulfiram 250 mg once daily. These can be started on day one. After seven days artesunate 50 mg once daily, captopril 50 mg once daily, itraconazole 200 mg once daily, ritonavir 400 mg once daily, and sertraline 100 mg once daily are started. On the three week check [day 21] drugs are brought to full doses, given below.

Aprepitant 80 mg twice daily.

Artesunate 50 mg twice daily.

Auranofin 3 mg twice daily.

Captopril 50 mg twice daily.

Celecoxib 400 mg twice daily

Disulfiram 250 mg twice daily.

Itraconazole 200 mg twice daily.

Ritonavir 400 mg twice daily.

Sertraline 100 mg twice daily.

#### Several practical recommendations

As part of CUSP9* we specify first-choice medicines for four common problems arising in the course of glioblastoma treatment generally. We specify these drugs to treat ancillary problems due to the high potential for drug-drug interactions when so many drugs are used at once as in CUSP9*. The four selected drugs are unlikely to interact pharmacokinetically or pharmacodynamically with the CUSP9* drugs. No other medicine, herbal preparation, over-the-counter medicine or nutritional supplement will be allowed, again to minimize the risk of drug-drug interaction.

i. Diarrhea can be treated with loperamide 2 mg ii bid. This dose can be increased or decresed as needed. Loperamide is not absorbed systemically, yet by stimulating gut opiate receptors effectively reduces the loose bowel movements some of the CUSP9* drugs can give.

ii. Anxiety or poor sleep should be treated with lorazepam 1 mg or 2 mg at hour of sleep. This can be increased or decreased as needed. Half this dose can be used several times during the day should daytime anxiety become a problem.

iii. If seizures occur levetiracetam 1 gram twice daily can be used, with down titration if side effects are encountered.

iv. Headache treatment with low-dose hydromorphone is allowed.

An overview of pharmacodynamic and pharmacokinetic data and interaction analysis of the CUSP9* drugs is given in Table 4. Ritonavir and aprepitant are also inducers of 3A4. CUSP9* uses some of the most potent hepatic [and small intestine] P-450 3A4 inhibitors, itraconazole and riotonavir [see Table 4.]. This necessitates particular strictness in avoidance of any herbal preparations as well as any drug treatment beyond those specified by the CUSP9* regimen and the four ancillary drugs allowed- loperamide for diarrhea, lorazepam for anxiety or insomnia, levetiracetam for seizures, low-dose hydromorphone for headache.

Strictest avoidance of any source of ethanol, even in such sources as wine vinegar or casseroles and grapefruit juice avoidance [a 3A^4^ inhibitor] will be the only two dietary restrictions. Coffee consumption is allowed but caution advised, as caffeine effects can be magnified by 1A2 inhibitors like disulfiram.

Severe alcohol intolerance is not listed as a disulfiram side effect in that severe intolerance of even slight amounts of alcohol is universal and the main effect of disulfiram.

### Edema, dexamethasone, & survival

The use of dexamethasone is common during the course of glioblastoma particularly in the perioperative reduction of brain edema, but greater dexamethasone use is a negative prognostic sign [285]. Dexamethasone or other related corticosteroid use contributes to disease morbidity and rarely, mortality. It is immunosuppressive [286], exacerbating the inherent, unmedicated, glioblastoma-related immunosuppression [287], Dexamethasone reduced *in vitro* temozolomide cytotoxicity in U87 [288] and T98G glioma cells [289]. Although dexamethasone has some anti-cancer activities in other cancers, and may have such at some stages of glioblastoma, we consider the sum of data indicates probable benefit from less, or ideally no, dexamethasone use. We have outlined how several of the CUSP9* medicines are expected to act together to reduce glioblastoma related edema and thereby reduce or eliminate the need for dexamethasone use in the course of glioblastoma treatment.

## CONCLUSION

We presented six themes important to cancer therapy generally that we apply to recurrent glioblastoma treatment and attempt to address by CUSP9*:

1. Nile Distributary Problem where cancer uses multiple cross-covering growth enhancing pathways to grow and avoid cytotoxic interventions.

2. Intratumoral heterogeneity in space and over time as general feature of cancer that must be accounted for in treatment.

3. Individual mutually supporting sub-populations existing within a tumor, working together to enhance growth, generating vigor of the malignant state.

4. Ganging up. We see the need for multiple attacks against the same growth-enhancing subsystem, again in the effort to defeat compensating reactions by tumors.

5. We must fight today with the weapons we have today. We therefore use ancillary attributes of nine already-marketed drugs to condition glioblastoma cells to be less fit to thrive.

6. “Changes across metabolic networks” that are not malignant by themselves become an integral part of malignant growth when combined with the suite of so deranged networks and genomic changes driving them. We aim to dismantle or block these pathologically employed but not inherently pathological processes enough to hobble glioblastoma growth.

We have shown how past research indicates how the nine drugs of CUSP9* have a good chance of inhibiting the growth enhancing functions of 17 different systems used by glioblastoma to grow, migrate, and avoid cell death. These growth systems are 1) AKT phosphorylation, 2) ALDH, 3) ACE, 4) carbonic anhydrase 2, 9, and 12, 5) COX-2, 6) cathepsin B, 7) Hh, 8) interleukin-6, 9] 5-LO, 10) MMP-2 and -9, 11) mTOR, 12) NK-1, 12) p-gp efflux pump, 14) thioredoxin reductase, 15) TF, 16) TCTP, and 17) VEGF.

Given the stalled progress in glioblastoma treatment since the last advance, introduction of temozolomide with the Stupp Protocol in 2005, we suggest a conceptual departure from the usual cytotoxic efforts that have so far been futile in prolonging survival or QOL. A formal clinical trial of CUSP9* in patients on first recurrence is in advanced stage planning. Initial experience on the handful of patients as reported to us who were given CUSP9 without remarkable side effects leads us to be optimistic that this will hold for the closely related CUSP9*.

To break the impasse we propose using nine commonly used drugs marketed for non-cancer indications, drugs that show minimal cytotoxicity to normal cells and minimal changes to organ systems when used singly. We use the nine drugs of CUSP9* to “shape the battlefield”. By this we mean if we can inhibit or block a pathological protective system engaged or used by glioblastoma cells to grow, migrate, or avoid cell death and senescence, our directly cell-killing drugs like temozolomide will be more effective. The nine drugs of CUSP9* each have data from animal and human study showing they can individually inhibit or block one or another identified cellular pathway to enhance or stimulate their growth, helping CUSP9* drugs acting in concert to set the stage for more effective cell killing.

## Abbreviations

ARB: angiotensin receptor blockers
ACE: angiotensin converting enzyme
ATR1: angiotensin II receptor 1
BBB: blood-brain barrier
BCRP: breast cancer resistance protein
COX: cyclooxygenase
CUSP9: Coordinated Undermining of Survival Paths
DMARDs: disease modifying anti-rheumatic drugs
EMA: European Medicines Agency
iNOS: inducible nitric oxide synthase
5-LO: 5-lipoxygenase
MCP-1: macrophage chemotactic protein-1, synonymous with CCL2
MMP: matrix metalloproteinase
MGMT: O6-methylguanine-DNA methyltransferase
NK-1: neurokinin-1 receptors
OS: overall survival
p-gp: P-glycoprotein, synonymous with MDR-1
PGE2: prostaglandin E2
RAS: renin-angiotensin system
QOL: quality of life
ROS: reactive oxygen species
RCC: renal cell carcinoma
TCTP: Translationally controlled tumor protein
TF: Tissue Factor, also termed thromboplastin
TNF-alpha: tumor necrosis factor-alpha
VEGF: vascular endothelial growth factor
